# Colonization Resistance: Battle of the Bugs or Ménage à Trois with the Host?

**DOI:** 10.1371/journal.ppat.1003730

**Published:** 2013-11-21

**Authors:** Alanna M. Spees, Christopher A. Lopez, Dawn D. Kingsbury, Sebastian E. Winter, Andreas J. Bäumler

**Affiliations:** Department of Medical Microbiology and Immunology, School of Medicine, University of California at Davis, Davis, California, United States of America; Duke University Medical Center, United States of America

The lower gastrointestinal tract is host to a dense microbial community, known as the gut microbiota, which is dominated by obligate anaerobic bacteria belonging to the phyla Bacteroidetes (class Bacteroidia) and Firmicutes (class Clostridia). This microbial community offers benefit by conferring niche protection against invading microbes, a property known as “colonization resistance” (reviewed in [1]). The concept of colonization resistance was initially derived from studies showing that an antibiotic-mediated disruption of gut-associated microbial communities greatly enhances the ability of facultative anaerobic Enterobacteriaceae, such as *Escherichia coli* or *Salmonella enterica*, to colonize the large bowel [2]–[4]. Conventional wisdom holds that resident Bacteroidia and Clostridia confer colonization resistance by “competitive exclusion” of Enterobacteriaceae (phylum Proteobacteria) through microbe-microbe interactions. However, recent studies draw a tantalizing new picture, which suggests that host interactions might play a central role in shaping the microbial community structure. Here we review these new hypotheses and their implications for human health.

## Microbe-Microbe Interactions and Conventional Wisdom

Microbe-microbe interactions are clearly important during the competition of bacterial species that occupy similar metabolic niches. For example, rivalry between closely related Proteobacteria arises from competition for carbon sources [5] or trace elements, such as iron [6]. Changes in the availability of nutrients have also been proposed as a mechanism for increasing the relative abundance of Proteobacteria within the community. This “food hypothesis” suggests that disruption of the community of obligate anaerobic Bacteroidia and Clostridia by antibiotic treatment increases the availability of high-energy nutrients, such as carbohydrates, which supports proliferation of fast-growing Proteobacteria [7]. Consistent with this idea, antibiotic treatment of mice increases the availability in the cecal lumen of the microbiota-liberated mucosal carbohydrates sialic acid and fucose, and the ability to consume these sugars confers a two-fold fitness advantage upon *S. enterica* during growth in the large bowel [8]. While these data show that elevated levels of mucus-derived carbohydrates lead to a two-fold rise in bacterial numbers, it is also clear that additional mechanisms might be at work because streptomycin treatment of mice increases the cecal recovery of *E. coli* and *S. enterica* by more than 1,000-fold [9].

**Figure 1:**
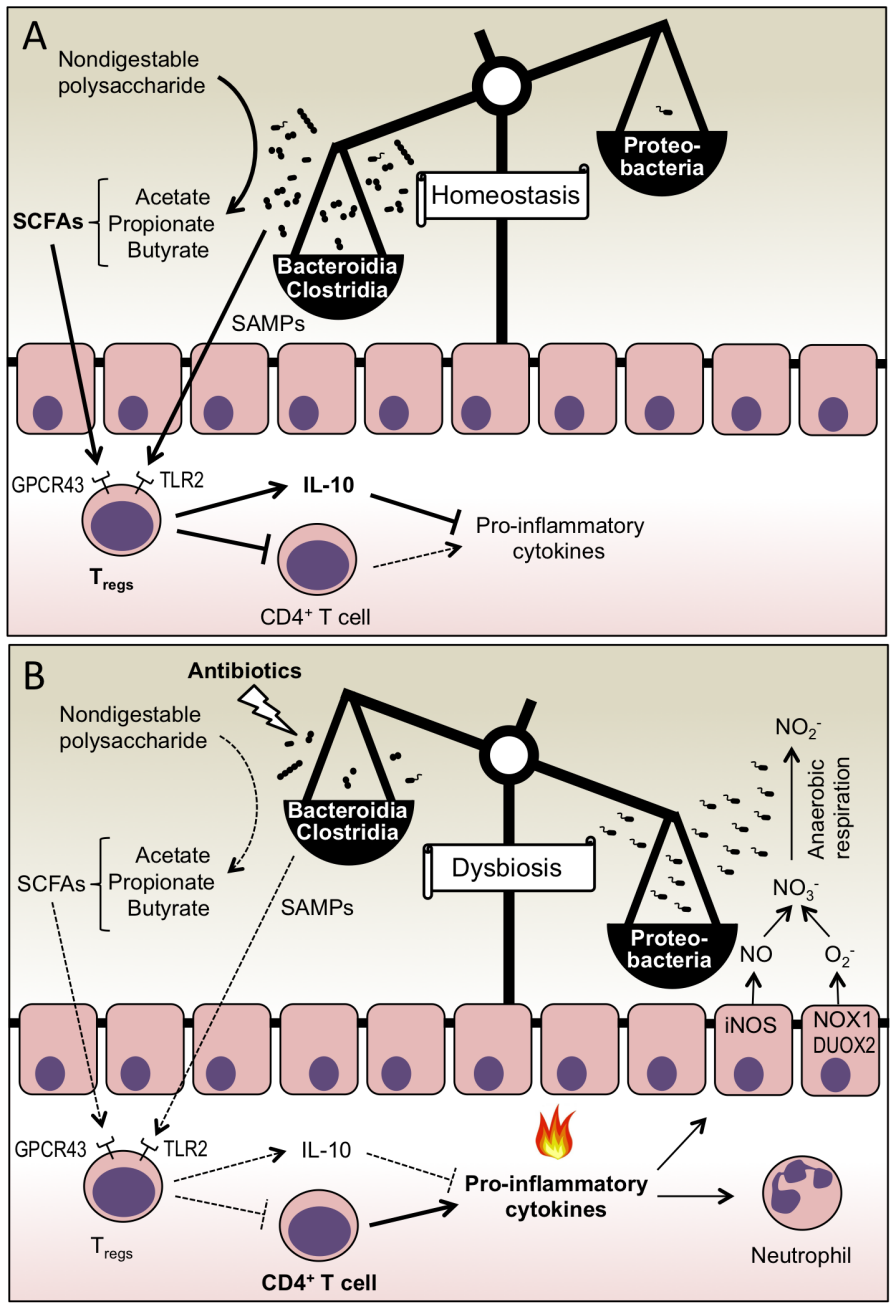
Colonization resistance against Proteobacteria is mediated by host-directed nutritional exclusion. (A) Microbe-host interactions maintain intestinal immune homeostasis. SCFAs and SAMPs produced by obligate anaerobic Clostridia and Bacteroidia stimulate T_regs_, which suppress inflammation by producing IL-10 and by limiting the expansion of CD4^+^ effector T cells. (B) Treatment with antibiotics disrupts microbial communities that produce SCFAs and SAMPs. The consequent increase in the inflammatory tone of the intestinal mucosa results in the activation of enzymes, such as iNOS, NADPH oxidase 1 (NOX1), and dual-function NADPH oxidase 2 (DUOX2), which generate reactive nitrogen species (NO) and reactive oxygen species (O_2_^−^). Respiratory electron acceptors (NO_3_^−^) generated as a by-product of the host response support growth of Proteobacteria by anaerobic respiration. The consequent bloom of Proteobacteria leads to an imbalance in the microbial community structure (dysbiosis).

Microbe-microbe interactions have furthermore been evoked in the metabolic exclusion of members of the phylum Proteobacteria by fermentation end products of distantly related bacteria belonging to the phyla Bacteroidetes and Firmicutes. This hypothesis is based on the observation that complex polysaccharides, which cannot be broken down in the upper gastrointestinal tract of mammals, are hydrolyzed and fermented in the large bowel into short-chain fatty acids (SCFAs) by obligate anaerobic bacteria, with Clostridia being credited for producing the lion's share (reviewed in [10]) (Figure 1A). In turn, SCFAs can reduce intestinal inflammation, which correlates with reduced numbers of colitogenic Proteobacteria in a mouse colitis model [11]. Furthermore, the presence of SCFAs is associated with reduced growth of *E. coli* or *S. enterica* under certain *in vitro* conditions. Based on these observations, production of SCFAs by obligate anaerobic bacteria is commonly assumed to be a mechanism for metabolic exclusion of Proteobacteria from the large bowel (reviewed in [1]). Nonetheless, recent data on the role of SCFAs during microbe-host interaction provide compelling support for an alternative interpretation of these data.

## Microbe-Host Interactions Maintain Immune Homeostasis

In addition to their proposed role during microbe-microbe interactions leading to metabolic exclusion, SCFAs influence several aspects of host cell signaling and metabolism. One important function of SCFAs is to provide nutrition for colonocytes, which utilize bacterially produced butyrate as their primary energy source [12]. Furthermore, SCFAs can activate host cell signaling by binding to G protein–coupled receptor 43 (GPCR43, also known as GPR43) [13], thereby altering host cell physiology. One consequence is that colonocytes oxidize carbon sources to CO_2_ when butyrate is present, but in the large bowel of germ-free mice where butyrate is absent their metabolism switches toward fermentative lactate production [12]. Finally, the evidence discussed below suggests that SCFAs contribute to the maintenance of immune homeostasis in the lower gastrointestinal tract.

Colonization of the large bowel with commensal microbial communities confers protection from inflammatory disease through mechanisms that are only just beginning to be elucidated. *Bacteroides fragilis*, a prominent human commensal, synthesizes polysaccharide A (PSA), a symbiont-associated molecular pattern (SAMP) that suppresses inflammatory responses in the intestine [14] by engaging TLR2 expressed on regulatory T cells (T_regs_) [15]. Interestingly, SCFAs produced by the gut microbiota suppress inflammation by a similar mechanism. SCFAs bind to GPCR43 expressed by colonic T_regs_. Upon stimulation with SCFAs, colonic T_regs_ expand and mediate resolution of inflammation by increasing expression of the anti-inflammatory cytokine IL-10 (interleukin-10) and by limiting proliferation of effector CD4^+^ T cells [16] (Figure 1A).

Disruption of the gut microbiota by vancomycin treatment reduces the levels of colonic T_regs_, but this reduction is completely restored when mice are treated with a combination of vancomycin and SCFAs [17]. The fact that vancomycin treatment reduces intestinal SCFA concentrations [16] and enhances the ability of *S. enterica* to colonize the gut [18] supports the concept that SCFAs play a role in mediating colonization resistance against Proteobacteria. However, these novel insights also suggest that there might be an alternative explanation for the underlying mechanism other than the microbe-microbe interaction (i.e., competitive exclusion) postulated previously. That is to say, SCFAs might contribute to colonization resistance indirectly through microbe-host interactions, which maintain colonic T_reg_ homeostasis. This new hypothesis suggests that antibiotics, by lowering SCFA concentrations, reduce the abundance and activation state of colonic T_regs_ [16], thereby increasing the inflammatory tone of the mucosa in the lower gastrointestinal tract (Figure 1B). But to explain a loss of colonization resistance after antibiotic treatment by this hypothesis, one has to consider the possibility that the ensuing inflammatory response somehow supports the growth of Proteobacteria. While this assumption has not been tested in studies reporting that SCFAs modulate host immune responses, a number of publications discussed below suggest that intestinal inflammation does, in fact, lower colonization resistance against Proteobacteria.

## Host-Microbe Interactions Feed Proteobacteria

The concept that host responses can reduce colonization resistance against Proteobacteria was first introduced by reports demonstrating that colitis elicited in response to an enteric pathogen, a chemical trigger, or genetic predisposition markedly increases the prevalence of Enterobacteriaceae within murine gut–associated microbial communities [19]–[21]. Consistent with this idea, a microbial imbalance (dysbiosis) characterized by an increased prevalence of Proteobacteria is observed during intestinal inflammatory disorders in humans, such as Crohn's disease [22], enteropathy in human immunodeficiency virus (HIV)-infected subjects [23], or necrotizing enterocolitis in preterm infants [24]. This enrichment for Proteobacteria during inflammation has been arguably the most consistent and robust ecological pattern observed in the mammalian lower gastrointestinal tract.

A host-microbe interaction responsible for a loss of colonization resistance against Proteobacteria was recently identified as the generation of respiratory electron acceptors, which are produced as a by-product of the host inflammatory response [25]. For example, iNOS (inducible nitric oxide synthase) and NADPH oxidases induced during inflammation produce nitric oxide (NO) and superoxide radicals (O_2_^−^), respectively, which can react to form nitrate (NO_3_^−^). In the anaerobic environment of the healthy large intestine, obligate anaerobic Clostridia and Bacteroidia have a competitive growth advantage over the facultative anaerobic Proteobacteria (reviewed in [26]). But by using host-derived nitrate as a terminal electron acceptor for anaerobic respiration, both commensal *E. coli* and the pathogen *S. enterica* serovar Typhimurium can bloom in the lumen of the inflamed lower gastrointestinal tract (Figure 1B) [27], [28]. The ability to respire nitrate or other electron acceptors generated as by-products of the host inflammatory response is widely conserved among the facultative anaerobic Proteobacteria. In contrast, the obligate anaerobic Clostridia and Bacteroidia lack the terminal oxidoreductases needed to use these exogenous electron acceptors (reviewed in [10]). As a result, Clostridia and Bacteroidia compete for a limited amount of fermentable carbohydrates and amino acids, while Proteobacteria can sidestep this competition during inflammation by using anaerobic respiration to grow on fermentation end products or on other non-fermentable carbon sources [29]. Through this mechanism, the host inflammatory response selectively feeds facultative anaerobic Proteobacteria, thereby increasing their prevalence within communities inhabiting the large bowel.

## How Antibiotics Lower Colonization Resistance

One remaining question is whether this mechanism contributes to the antibiotic-induced reduction in colonization resistance against Proteobacteria first reported in 1954 [2]. The recent discovery that a disruption of the gut microbiota by vancomycin lowers intestinal SCFA concentrations and reduces colonic T_regs_ [16], [17] suggests that antibiotics might increase the inflammatory tone of the mucosa in the large bowel. Consistent with this prediction, mild infiltrates of inflammatory monocytes, neutrophils, and natural killer cells are observed in the cecal mucosa of mice treated orally with metronidazole or streptomycin [9], [30]. More importantly, streptomycin treatment markedly increases expression of *NOS2*, the gene encoding iNOS, in the cecal mucosa. The consequent streptomycin-induced generation of host-derived nitrate boosts luminal growth of *E. coli* by anaerobic respiration [9]. Collectively, these publications strongly support the idea that antibiotics lower colonization resistance against Proteobacteria by increasing the inflammatory tone of the intestinal mucosa (Figure 1B).

## Assembling the Bigger Picture

The recent publications reviewed above have each contributed key pieces to a puzzle that can now, for the first time, be assembled into a coherent model. The emerging image suggests that colonization resistance against Proteobacteria is mediated by microbe-host interactions that maintain intestinal immune homeostasis (Figure 1A). Antibiotics can diminish this microbe-host interaction by disrupting the microbial community of obligate anaerobic SCFA producers [16]. Through this mechanism, antibiotics can increase the inflammatory tone of the intestinal mucosa, thereby initiating host-microbe interactions that selectively feed Proteobacteria (Figure 1B) [9], [27]. Further work is needed to test this hypothesis, for instance by determining whether a disruption of SCFA-mediated immune homeostasis is indeed responsible for the antibiotic-induced loss of colonization resistance against Proteobacteria. Should such studies provide support for the model depicted in Figure 1B, this would establish the concept that colonization resistance involves a ménage à trois between obligate anaerobic bacteria, the host immune system, and Proteobacteria. We propose the term “host-directed nutritional exclusion” to differentiate this novel concept from the preceding image of microbe-microbe interactions, which is commonly described as “competitive exclusion” or “metabolic exclusion”.”

Furthermore, the proposed model (Figure 1B) might have significant implications for understanding the pathogenesis of irritable bowel syndrome (IBS), a condition characterized by low-level intestinal inflammation and diarrhea, which often follows surgery or repeated courses of antibiotics. IBS patients have a lower concentration of total SCFAs [31], [32], and their fecal microbial population is characterized by an increased abundance of Proteobacteria [33]–[37]. The streptomycin-treated mouse exhibits many features that are characteristic of human IBS and might thus represent a useful animal model for exploring the therapeutic value of approaches aimed at increasing SCFA concentrations for treatment.

## References

[1] Lawley TD, Walker AW (2013) Intestinal colonization resistance. Immunology 138: 1–11. 23240815 10.1111/j.1365-2567.2012.03616.x PMC3533696

[2] Bohnhoff M, Drake BL, Miller CP (1954) Effect of streptomycin on susceptibility of intestinal tract to experimental *Salmonella* infection. Proc Soc Exp Biol Med 86: 132–137. 13177610 10.3181/00379727-86-21030

[3] Saito K (1961) Studies on the habitation of pathogenic *Escherichia coli* in the intestinal tract of mice. I. Comparative experiments on the habitation of each type of resistant pathogenic *Escherichia coli* under an administration of streptomycin. Paediatria Japonica 65: 385–393. 13745460

[4] Saito K (1961) Studies on the habitation of pathogenic *Escherichia coli* in the intestinal tract of mice. II. Experimental inoculation of type 055 *Escherichia coli* after long-term administration of streptomycin. Paediatria Japonica 65: 394–399. 13745461

[5] Maltby R, Leatham-Jensen MP, Gibson T, Cohen PS, Conway T (2013) Nutritional basis for colonization resistance by human commensal *Escherichia coli* strains HS and Nissle 1917 against *E. coli* O157:H7 in the mouse intestine. PLoS ONE 8: e53957 doi:10.1371/journal.pone.0053957 23349773 10.1371/journal.pone.0053957 PMC3547972

[6] Deriu E, Liu JZ, Pezeshki M, Edwards RA, Ochoa RJ, et al (2013) Probiotic bacteria reduce *Salmonella typhimurium* intestinal colonization by competing for iron. Cell Host Microbe 14: 26–37. 23870311 10.1016/j.chom.2013.06.007 PMC3752295

[7] Stecher B, Hardt WD (2008) The role of microbiota in infectious disease. Trends Microbiol 16: 107–114. 18280160 10.1016/j.tim.2007.12.008

[8] Ng KM, Ferreyra JA, Higginbottom SK, Lynch JB, Kashyap PC, et al (2013) Microbiota-liberated host sugars facilitate post-antibiotic expansion of enteric pathogens. Nature 502: 96–99. 23995682 10.1038/nature12503 PMC3825626

[9] Spees AM, Wangdi T, Lopez CA, Kingsbury DD, Xavier MN, et al (2013) Streptomycin-induced inflammation enhances *Escherichia coli* gut colonization through nitrate respiration. mBio 4: e00430–13. 10.1128/mBio.00430-13 PMC3705454 23820397

[10] Fischbach MA, Sonnenburg JL (2011) Eating for two: how metabolism establishes interspecies interactions in the gut. Cell Host Microbe 10: 336–347. 22018234 10.1016/j.chom.2011.10.002 PMC3225337

[11] Veiga P, Gallini CA, Beal C, Michaud M, Delaney ML, et al (2010) *Bifidobacterium animalis* subsp. lactis fermented milk product reduces inflammation by altering a niche for colitogenic microbes. Proc Natl Acad Sci U S A 107: 18132–18137. 20921388 10.1073/pnas.1011737107 PMC2964251

[12] Donohoe DR, Wali A, Brylawski BP, Bultman SJ (2012) Microbial regulation of glucose metabolism and cell-cycle progression in mammalian colonocytes. PLoS ONE 7: e46589 doi:10.1371/journal.pone.0046589 23029553 10.1371/journal.pone.0046589 PMC3460890

[13] Nilsson NE, Kotarsky K, Owman C, Olde B (2003) Identification of a free fatty acid receptor, FFA2R, expressed on leukocytes and activated by short-chain fatty acids. Biochem Biophys Res Commun 303: 1047–1052. 12684041 10.1016/s0006-291x(03)00488-1

[14] Mazmanian SK, Round JL, Kasper DL (2008) A microbial symbiosis factor prevents intestinal inflammatory disease. Nature 453: 620–625. 18509436 10.1038/nature07008

[15] Round JL, Lee SM, Li J, Tran G, Jabri B, et al (2011) The Toll-like receptor 2 pathway establishes colonization by a commensal of the human microbiota. Science 332: 974–977. 21512004 10.1126/science.1206095 PMC3164325

[16] Smith PM, Howitt MR, Panikov N, Michaud M, Gallini CA, et al (2013) The microbial metabolites, short-chain fatty acids, regulate colonic Treg cell homeostasis. Science 341: 569–573. 23828891 10.1126/science.1241165 PMC3807819

[17] Atarashi K, Tanoue T, Shima T, Imaoka A, Kuwahara T, et al (2011) Induction of colonic regulatory T cells by indigenous Clostridium species. Science 331: 337–341. 21205640 10.1126/science.1198469 PMC3969237

[18] Sekirov I, Tam NM, Jogova M, Robertson ML, Li Y, et al (2008) Antibiotic-induced perturbations of the intestinal microbiota alter host susceptibility to enteric infection. Infect Immun 76: 4726–4736. 18678663 10.1128/IAI.00319-08 PMC2546810

[19] Lupp C, Robertson ML, Wickham ME, Sekirov I, Champion OL, et al (2007) Host-mediated inflammation disrupts the intestinal microbiota and promotes the overgrowth of Enterobacteriaceae. Cell Host Microbe 2: 119–129. 18005726 10.1016/j.chom.2007.06.010

[20] Stecher B, Robbiani R, Walker AW, Westendorf AM, Barthel M, et al (2007) *Salmonella enterica* serovar Typhimurium exploits inflammation to compete with the intestinal microbiota. PLoS Biol 5: e244 doi:10.1371/journal.pbio.0050244 10.1371/journal.pbio.0050244 PMC1951780 17760501

[21] Barman M, Unold D, Shifley K, Amir E, Hung K, et al (2008) Enteric salmonellosis disrupts the microbial ecology of the murine gastrointestinal tract. Infect Immun 76: 907–915. 18160481 10.1128/IAI.01432-07 PMC2258829

[22] Frank DN, St Amand AL, Feldman RA, Boedeker EC, Harpaz N, et al (2007) Molecular-phylogenetic characterization of microbial community imbalances in human inflammatory bowel diseases. Proc Natl Acad Sci U S A 104: 13780–13785. 17699621 10.1073/pnas.0706625104 PMC1959459

[23] Vujkovic-Cvijin I, Dunham RM, Iwai S, Maher MC, Albright RG, et al (2013) Dysbiosis of the gut microbiota is associated with HIV disease progression and tryptophan catabolism. Sci Transl Med 5: 193ra191. 10.1126/scitranslmed.3006438 PMC4094294 23843452

[24] Normann E, Fahlen A, Engstrand L, Lilja HE (2013) Intestinal microbial profiles in extremely preterm infants with and without necrotizing enterocolitis. Acta paediatrica 102: 129–136. 23082780 10.1111/apa.12059

[25] Winter SE, Thiennimitr P, Winter MG, Butler BP, Huseby DL, et al (2010) Gut inflammation provides a respiratory electron acceptor for *Salmonella* . Nature 467: 426–429. 20864996 10.1038/nature09415 PMC2946174

[26] Winter SE, Lopez CA, Bäumler AJ (2013) The dynamics of gut-associated microbial communities during inflammation. EMBO Rep 14: 319–327. 23478337 10.1038/embor.2013.27 PMC3615657

[27] Winter SE, Winter MG, Xavier MN, Thiennimitr P, Poon V, et al (2013) Host-derived nitrate boosts growth of *E. coli* in the inflamed gut. Science 339: 708–711. 23393266 10.1126/science.1232467 PMC4004111

[28] Lopez CA, Winter SE, Rivera-Chavez F, Xavier MN, Poon V, et al (2012) Phage-mediated acquisition of a type III secreted effector protein boosts growth of *Salmonella* by nitrate respiration. mBio 3: e00143–12. 22691391 10.1128/mBio.00143-12 PMC3374392

[29] Thiennimitr P, Winter SE, Winter MG, Xavier MN, Tolstikov V, et al (2011) Intestinal inflammation allows *Salmonella* to use ethanolamine to compete with the microbiota. Proc Natl Acad Sci U S A 108: 17480–17485. 21969563 10.1073/pnas.1107857108 PMC3198331

[30] Wlodarska M, Willing B, Keeney KM, Menendez A, Bergstrom KS, et al (2011) Antibiotic treatment alters the colonic mucus layer and predisposes the host to exacerbated *Citrobacter rodentium*-induced colitis. Infect Immun 79: 1536–1545. 21321077 10.1128/IAI.01104-10 PMC3067531

[31] Kopecny J, Simunek J (2002) Cellulolytic bacteria in human gut and irritable bowel syndrome. Acta Veterinaria Brno 71: 421–427.

[32] Treem WR, Ahsan N, Kastoff G, Hyams JS (1996) Fecal short-chain fatty acids in patients with diarrhea-predominant irritable bowel syndrome: in vitro studies of carbohydrate fermentation. J Pediatr Gastroenterol Nutr 23: 280–286. 8890079 10.1097/00005176-199610000-00013

[33] Matto J, Maunuksela L, Kajander K, Palva A, Korpela R, et al (2005) Composition and temporal stability of gastrointestinal microbiota in irritable bowel syndrome–a longitudinal study in IBS and control subjects. FEMS Immunol Med Microbiol 43: 213–222. 15747442 10.1016/j.femsim.2004.08.009

[34] Krogius-Kurikka L, Lyra A, Malinen E, Aarnikunnas J, Tuimala J, et al (2009) Microbial community analysis reveals high level phylogenetic alterations in the overall gastrointestinal microbiota of diarrhoea-predominant irritable bowel syndrome sufferers. BMC Gastroenterol 9: 95. 20015409 10.1186/1471-230X-9-95 PMC2807867

[35] Saulnier DM, Riehle K, Mistretta TA, Diaz MA, Mandal D, et al (2011) Gastrointestinal microbiome signatures of pediatric patients with irritable bowel syndrome. Gastroenterology 141: 1782–1791. 21741921 10.1053/j.gastro.2011.06.072 PMC3417828

[36] Kerckhoffs AP, Ben-Amor K, Samsom M, van der Rest ME, de Vogel J, et al (2011) Molecular analysis of faecal and duodenal samples reveals significantly higher prevalence and numbers of *Pseudomonas aeruginosa* in irritable bowel syndrome. J Med Microbiol 60: 236–245. 20947663 10.1099/jmm.0.022848-0

[37] Carroll IM, Ringel-Kulka T, Siddle JP, Ringel Y (2012) Alterations in composition and diversity of the intestinal microbiota in patients with diarrhea-predominant irritable bowel syndrome. Neurogastroenterol Motil 24: 521–530, e248. 22339879 10.1111/j.1365-2982.2012.01891.x PMC3975596

